# Ketone Bodies in the Regulation of Myocardial Perfusion in Cardiovascular Disease: Metabolic and Vasodilatory Effects

**DOI:** 10.3390/ijms26104856

**Published:** 2025-05-19

**Authors:** Afolasayo A. Aromiwura, Kara R. Gouwens, Daniel C. Nguyen, Maryta Sztukowska, Luanne Didelot, Dinesh K. Kalra

**Affiliations:** 1 Division of Cardiology, Department of Medicine, University of Louisville, Louisville, KY 40202, USA; 2 Department of Medicine, Christina Lee Brown Envirome Institute, Center for Cardiometabolic Science, University of Louisville, Louisville, KY 40202, USAdaniel.nguyen.1@louisville.edu (D.C.N.); 3 Department of Medicine, College of Nursing, University of Information Technology and Management, 35-225 Rzeszów, Poland; 4 Clinical Trials Unit, School of Medicine, University of Louisville, Louisville, KY 40202, USA

**Keywords:** ketone bodies, myocardial blood flow, metabolic vasodilation

## Abstract

Ketone bodies (KBs) serve as an alternative energy source for healthy and failing hearts and have important effects on myocardial blood perfusion in both physiological and pathological states. Early animal studies suggest that KBs may provide protective benefits in ischemic heart disease and heart failure. Under normal circumstances, coronary blood flow regulation is an intricate system with contributions from metabolic, autonomic, compressive, and endothelial factors, with the metabolic regulatory pathway being the most significant contributor. We conducted a non-systematic review of studies published between 1987 and 2024. In this review, we explored the physiological autoregulation of normal coronary blood flow, the role of ketone bodies in myocardial perfusion in health and disease, and the potential role of exogenous ketone body supplementation in producing salutary effects on myocardial blood flow (MBF) and metabolism in exercise and cardiac disease states including ischemia, heart failure, and the aging heart. Overall, our findings demonstrated that KBs improve MBF and ejection fraction in healthy human subjects and have beneficial effects on cardiac output and left heart filling pressures in patients with decompensated heart failure. Although resting myocardial blood flow decreases with age, further studies are required to assess the impact of KBs on MBF in aging populations. Additionally, more research is needed to investigate the effects of KBs during exercise and in instances of myocardial ischemia.

## 1. Introduction

A hallmark of many cardiovascular diseases (CVDs) is inadequate myocardial oxygen delivery, where vasodilatory processes and myocardial blood flow become uncoupled from cardiac workload and myocardial volume oxygen consumption (MVO_2_) [1]. In fact, coronary microvascular dysfunction often precedes left ventricular (LV) remodeling and overt symptomatic ischemic heart disease (IHD) or heart failure (HF). Non-invasive coronary disease treatment involves risk factor modification and drugs such as aspirin, lipid-lowering medications, beta-blockers, and RAAS blockade. These drugs act via multiple pathways, including anti-inflammatory, anti-fibrotic, anti-platelet, anti-atherosclerosis, reverse remodeling, and vasodilatory effects [2,3,4,5]. Concurrently, under normal conditions, there is a robust and responsive blood flow–metabolic regulatory axis where changes in local cardiac metabolism are met with near-instant changes in myocardial blood flow (MBF) [6]. As such, the uncoupling of MBF to cardiac work in various CVDs, including IHD, could be partially explained by the impaired MBF and reprogrammed cardiac metabolism.

In particular, most forms of heart failure are associated with the uncoupling of the mitochondrial oxidation pathway with ATP generation and a preferential shift from fatty acids toward glucose utilization for oxidation [7]. Meanwhile, ketone bodies (KBs) can serve as an alternative fuel source for the heart. In many cardiac diseases, the normal substrate of free fatty acid (FFA) uptake can be altered such that the heart may utilize glucose, amino acids, or KBs under stress conditions, thereby changing its metabolism to a pro-perfusion phenotype [8]. In line with these observations, new strategies of delivering KBs as a potential therapeutic intervention for heart failure have been shown to improve cardiac function and increase coronary perfusion, signifying improved coupling between changes in cardiac workload/MVO_2_ with coronary perfusion [9].

KBs may not only have a role as a therapeutic in CVD but have also been found to increase cardiac output/perfusion in healthy hearts [9]. Thus, there is ongoing research on using ketone bodies to prevent cardiac decline and enhance exercise capacity [10]. Here, we discussed the physiological regulation of coronary blood flow and myocardial perfusion and the potential coupling with KBs that may augment MBF in diseased or increased myocardial demand states such as IHD, heart failure (HF), exercise, and aging.

## 2. Methodology

To inform the selection process, we searched PubMed for English language original articles published between 1987 and 2025. We reviewed approximately 50 articles, and relevant materials were included in our review.

## 3. Physiological Regulation of Myocardial Blood Flow in the Healthy Heart

In healthy hearts, baseline oxygen and nutrient extraction from the LV coronary circulation is incredibly efficient at approximately 60–80%. Therefore, increased cardiac stress must be matched with appropriate increases in MBF to meet metabolic demand [11]. This high extraction rate is due to the dense capillary beds located in the LV. However, the dense capillary networks in the LV have limited capillary reserve recruitment [12]. Instead, the heart must rely on increased MBF to meet energetic demands (myocardial hyperemia) [13]. This hyperemic response of the heart is essential to supporting it under increased stress, such as exercise.

When there is a mismatch between oxygen and nutrient supply and demand (an impaired hyperemic response), the heart cannot sustain higher cardiac work. Impaired cardiac work limits capacity and can result in myocardial ischemia and heart failure over time [13,14,15]. Unsurprisingly, microvascular dysfunction in the heart is often an early sign and prognostic indicator of diseases such as myocardial infarction with non-obstructive coronary arteries [16]. Lifestyle factors such as smoking, sedentary behavior, and sleep deprivation also contribute to impaired microvascular function [16,17,18]. Furthermore, research has shown that myocardial hyperemia is impaired with aging, underscoring the importance of cardiac perfusion for long-term heart health [17]. Thus, understanding the underlying regulation of MBF is essential for maintaining and treating cardiovascular health. In the following section, we will discuss the underlying physiology, prevailing theories of regulation, and ketone bodies as a therapeutic intervention for myocardial perfusion.

### 3.1. Autoregulation

Autoregulation is the dynamic process by which the coronary vessels adjust their tone in response to changes in perfusion pressure, thereby maintaining a relatively constant blood flow. This ensures that the supply of oxygen and nutrients matches the myocardium’s metabolic demands. The autoregulatory capacity of the LV operates within a perfusion pressure range of approximately 40 to 130 mmHg in healthy individuals. In this pressure range, changes in perfusion pressure produce a direct but transient change in coronary flow, followed by a return to baseline shortly after. The lower end of autoregulation is defined as the point at which the vessel has reached its maximum dilatory capacity; below this pressure, MBF becomes pressure-dependent. Several mechanisms contribute to this vital process.

**Figure 1:**
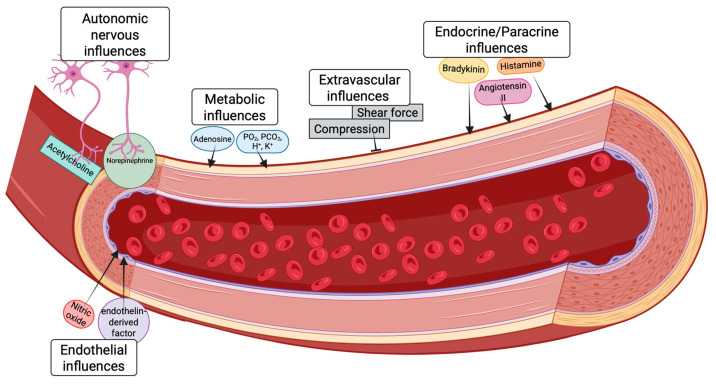
Regulators of coronary perfusion. Various autonomic, metabolic, endocrine, endothelial, and extravascular factors regulate coronary artery tone. H^+^—hydrogen ion, K^+^—potassium, PCO_2_—arterial or venous carbon dioxide, PO_2_—arterial or venous oxygen.

Originally, coronary perfusion autoregulation was thought to be solely under myogenic control but has since expanded to include endothelial, autonomic, metabolic, and endocrine control as mediators of autoregulation in the heart (Figure 1) [19,20].

Looking briefly at how each of these factors contribute to autoregulation, myogenic control is a key component of coronary autoregulation. Under myogenic control, an increase in intraluminal pressure stretches the vascular smooth muscle (VSM) cells in the arteriolar walls, leading to their depolarization and subsequent contraction (vasoconstriction), which reduces blood flow. Conversely, a decrease in pressure causes relaxation (vasodilation), increasing blood flow. This response is inherent to the smooth muscle cells themselves and can occur independently of the endothelium.

Endothelial regulation has been found to be involved in the expansion of the autoregulation perfusion pressure range, as the inhibition of nitric oxide synthase increases the lower limit of perfusion pressure without altering MBF [21].

Neural modulation by the autonomic nervous system is generally considered to have a weaker direct effect on coronary autoregulation compared to metabolic and myogenic mechanisms. This is because increased metabolism following neural stimulation, such as changes in heart rate and contractility, often overshadows direct vascular responses. Nevertheless, the metabolic consequence of neural stimulation is a significant mediator of the myocardial hyperemic response, with up to 25% of the hyperemic response being attributed to the direct activation of β-adrenergic receptors (β-AR) on cardiomyocytes [22].

Finally, metabolic control has become one of the prevailing theories of MBF autoregulation under normal perfusion pressure range, as changes in MVO_2_ have a nearly linear, direct correlation with MBF [23]. The metabolic mechanism proposes that a reduction in myocardial oxygen tension, resulting from decreased perfusion pressure, leads to the production and release of vasodilator metabolites (e.g., adenosine, carbon dioxide, potassium ions, hydrogen ions, reactive oxygen species). These metabolites then act on the coronary microvasculature to cause vasodilation, thereby restoring blood flow to the myocardium.

Disentangling the competing and parallel roles of myogenic behavior and metabolic control of autoregulation has proven particularly challenging, as both utilize Ca_v_1.2 channels on the VSM as their primary end-effector [24]. Consequently, metabolic and myogenic control are supported by a demonstration that inhibition of Ca_v_1.2 channels abolishes autoregulatory behavior [23]. Studies have attempted to parse out the influence of metabolic and myogenic control by comparing coronary response to hemodilution and hypoxemia, both of which augment blood flow, but the former challenge has little to no effect on venous PO_2_, while the latter significantly decreases venous PO_2_. These studies found that there must be both an adequate myogenic tone and an attained venous PO_2_ threshold (<25 mmHg) before a high degree of autoregulatory behavior is observed [24,25,26]. However, more work needs to be undertaken to understand the interplay between metabolic and myogenic control of autoregulation. More detailed discussions of autoregulation can be found in the works of Goodwill et al., Duncker et al., Johnson et al., and Tune et al. [6,20,24,27].

### 3.2. Metabolic Mechanisms of Autoregulatory Behavior

As stated above, metabolic regulation of MBF has become one of the prevailing theories of coronary flow autoregulation. This hypothesis currently theorizes that myocardial oxygen tension mediates autoregulation by increasing vasoactive metabolism as perfusion pressure decreases. In support of this, although the LV is under a higher compressive force than the RV, this does not result in a lower absolute MBF under resting conditions (LV: ~0.5–1.0 L mL/min/g; RV: ~0.3–0.6 mL/min/g), which is theorized to be due to differences in oxidative metabolism between LV and RV (LV: ~50–100 μL/O_2_/min; RV: ~30–50 μL/O_2_/min) [6]. MVO_2_ has also been shown to be tightly correlated with changes in MBF, specifically within the autoregulatory pressure range. As MVO_2_ changes based on the type of substrate (free fatty acids, glucose, lactate, ketones, etc.), the local substrate used for oxidative metabolism in the heart could serve as an important regulator of MBF [28]. In support of the importance of local substrate availability in the heart, during exercise, there is increased utilization of free fatty acids and lactate by the heart along with a decrease in glucose utilization [29]. Meanwhile, in certain forms of cardiovascular disease, not only does fatty acid oxidation become less efficient due to uncoupling from the electron transport chain, but the heart also increases the utilization of glucose for oxidative metabolism [30]. This concept has been further demonstrated in the working heart, where whenever cardiac work was held constant, changes in substrate utilization (free fatty acid or glucose) were sufficient to drive changes in MBF (palmitate: 12.3 ± 0.3; glucose: 9.45 ± 0.24 mL/min/g wet wt) with a concurrent change in O_2_ consumption (palmitate: 7.06 ± 0.08; glucose: 6.08 ± 0.13 μmol/min/g wet wt) [26,31,32]. These results have led to the speculation that the correlation between MVO_2_ and coronary flow may be from alterations in specific substrate utilization rather than MVO_2_ itself. In line with this updated theory, substrate utilization changes in states of improved (exercise) or impaired (cardiovascular disease and aging) microvascular function [7,29,30].

Looking at how cardiomyocyte substrate catabolism may be linked with vasomotion, we know that the Ca_v_1.2 channels in the smooth muscle are a critical endpoint for maintaining autoregulation, but there is a growing body of evidence showing K_v_ channels as an important regulator of Ca_v_1.2 channels due to studies demonstrating their sensitivity to both the redox and oxygenation state. Thus, K_v_ channels may serve to link cardiomyocyte metabolism to vascular tone [23,33,34]. This theory is further supported by the fact that changes in the substrate being oxidized in the working heart lead to altered redox potential, where constant rate infusion increased NAD^+^/NADH ratio from 2.15 ± 0.14 with palmitate infusion to 2.99 ± 0.57 with glucose supplementation [31]. In further support of K_v_ channels being an important link between metabolic changes and MBF, when K_v_ channels are inhibited, there is a reduction in coronary perfusion pressure from 40 to 140 mmHg but no change in autoregulatory behavior [23]. Additionally, changes in substrate uptake can lead to varying production of known vasodilators such as lactate, adenosine, and hydrogen peroxide. Thus, understanding how substrates used by the heart impact blood flow may lead to further delineation of the link between cardiomyocyte metabolism and VSM vasomotion, allowing us to optimize substrate supplementation to maintain a pro-perfusion phenotype.

When examining the substrates used in the ATP oxidative phosphorylation pathway in the heart, it has been observed that the heart uses free fatty acids for 40–60% of ATP production, glucose at 20–30%, ketone bodies at 6–7%, branched-chain amino acids at 4–5%, and lactate <3% (in the healthy heart). Interestingly, fatty acids (Oleate, Acetate), lactate, glucose, and acetoacetate are all sufficient to maintain cardiac function in the working heart, but beta-hydroxybutyrate (bOHB) was not unless additional glucose was present [35]. In vivo studies have demonstrated that infusion of exogenous bOHB results in increased MBF and enhanced cardiac performance without acting as a direct vasodilator at physiological concentrations [9,36]. Interestingly, a study in isolated mitochondria from the heart saw that the addition of bOHB had minimal impact on oxygen flux but resulted in more negative membrane potential and increased the ratio of NAD(P)H/NAD(P)^+^. Additionally, this study demonstrated that changes in mitochondrial energetics in response to bOHB were abolished following β-hydroxybutyrate dehydrogenase (*Bdh1*, BDH1), a redox-sensitive enzyme that converts bOHB to acetoacetate, deletion [37]. Additional work by Gouwens et al. has demonstrated that knocking out BDH1 in the cardiomyocyte attenuated the increase in MBF observed with acute hyperketonemia [38]. Thus, investigating how the bOHB influences MBF may provide a novel way to interrogate the metabolic regulation of vascular tone.

### 3.3. Ketone Bodies and Ketogenesis

**Figure 2:**
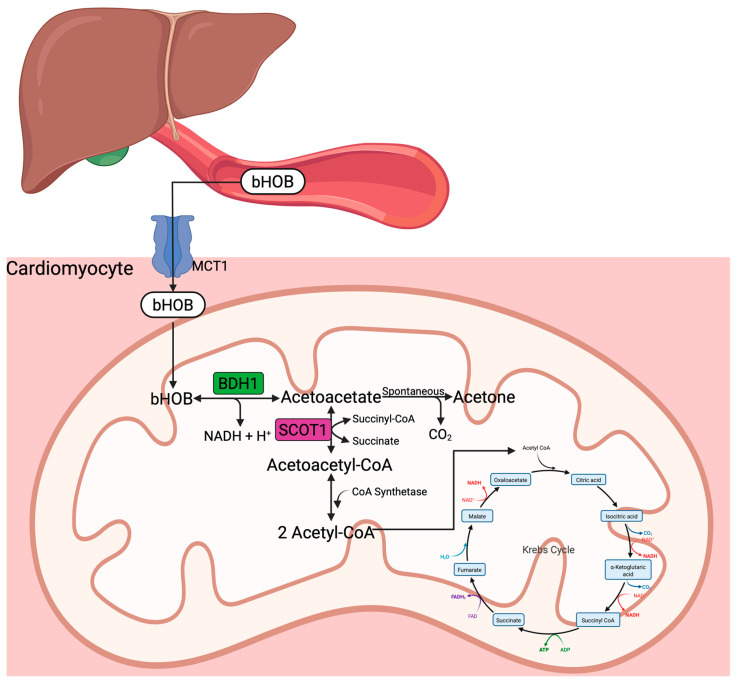
Schematic of ketone body oxidation. In the cardiomyocyte, bOHB is transported through the MCT and converted to acetoacetate, which may spontaneously form acetone or be converted to acetoacetyl-CoA by SCOT. Acetoacetyl-CoA forms 1 acetyl-CoA molecules that are oxidized in the Krebs cycle. bHOB—beta-hydroxybutyrate, BDH1—beta-hydroxybutyrate dehydrogenase 1, CO_2_—carbon dioxide, MCT1—monocarboxylate transporter, NADH—nicotinamide adenine dinucleotide reduced, SCOT1—succinyl-CoA:3-oxoacid-CoA transferase 1.

KBs, including bOHB, acetoacetate, and acetone, are mainly generated in the liver and derived from FFA oxidation in low glucose states. Acetyl-CoA generated from FFA oxidation in hepatocellular mitochondria is converted to HMG-CoA, a precursor of acetoacetate. The KBs synthesized in the liver are then transported through the bloodstream to their target organs, where they enter the cells via the monocarboxylate transporters (MCTs) (Figure 2). After entering the target cells, bOHB is transported into the mitochondria and converted to acetoacetate by bOHB dehydrogenase (*BDH1*). Subsequently, acetoacetate may undergo one of three fates: (1) reduction back to bOHB, (2) spontaneous decarboxylation to acetone, or (3) conversion to acetoacetyl-CoA via succinyl-CoA:3-oxoacid-CoA transferase (SCOT1). Acetoacetyl-CoA is cleaved into two acetyl-CoA molecules and utilized in the TCA cycle. Interestingly, BDH1 and SCOT1 are upregulated in the failing heart, and BDH1 is upregulated in the aging heart; thus, these hearts are primed to oxidize KBs as a fuel source upon their availability [39].

Additionally, SCOT1 is most concentrated in the heart compared to other organs, emphasizing that the heart is not only primed to use KBs under disease states but can also shift towards ketolysis in the failing heart [40,41,42]. There are several reasons for the heart to prioritize ketone utilization under duress. First, KB uptake into cardiomyocytes is minimally regulated, as circulating concentrations are low at baseline and rise in response to dietary or disease states, ranging from <0.1 mM, 2 mM, 6–8 mM, and 10–15 mM in the fed state, fasting/exercise, prolonged starvation, and diabetic ketoacidosis, respectively [36]. Therefore, cardiac KB oxidation is proportional to its circulating concentration and delivery, readily serving as an alternative fuel source for the heart under increased energetic demands. Another reason the diseased heart becomes primed for KB utilization is the myriad nonoxidative functions of bOHB, such as eliciting anti-inflammatory effects, reducing oxidative stress, and epigenetically modifying DNA histones [43,44]. Finally, as bOHB is taken up by the cardiomyocyte, metabolic reprogramming occurs, reducing glycolysis and alleviating the bottleneck in fatty acid oxidation [45].

Interestingly, KBs cannot be utilized in isolation, as their cataplerotic effects necessitate the use of anaplerotic substrates like glucose and glycogen, which replenish the Krebs cycle intermediates. Early studies on KB metabolism demonstrated that the perfusion of only KBs in isolated hearts resulted in acute contractile failure, partially due to the inhibition of the TCA cycle enzyme, 2-oxoglutarate dehydrogenase [46,47]. Conversely, in vivo bOHB administration has shown marked improvement in contractility and cardiac performance in both healthy and diseased states.

There are several ways to achieve hyperketonemia beyond the endogenous generation of KBs: ketone esters, ketone infusions, ketogenic diets, and SGLT2 (Sodium–glucose cotransporter 2) inhibition [41]. Increased circulating KB (~3 mM) has been found to improve cardiac function and increase myocardial perfusion in healthy and diseased hearts, signifying that the metabolic shifts that occur with bOHB infusion are pro-perfusion [48]. In the following section, we will discuss the current literature regarding the potential of hyperketonemia as a perfusion-enhancing factor and its subsequent potential as a therapeutic and exercise-enhancing strategy.

## 4. Influence of Ketone Bodies on Myocardial Perfusion

### 4.1. Clinical Findings

As the cardiac benefits of hyperketonemia are being discovered, this has led to the rapid expansion of research investigating the effects of KBs on cardiac function. A review of the SGLT-2 inhibitors theorized that one of the major benefits of bOHB may be from increased myocardial perfusion in both healthy human subjects and diseased states associated with hyperketonemia (Table 1).

Reviewing what is known about KB’s impact on MBF, a study involving eight healthy participants (median age, 60 years) tested the impact of sodium bOHB infusion versus saline infusion on myocardial glucose uptake and blood flow over two 390 min sessions [8]. Ketone infusion resulted in a 50% reduction of myocardial glucose uptake (304 nmol per gram per minute with saline vs. 156 nmol per gram per minute with ketone; *p* < 0.01), a 75% increase in MBF, and a 25% rise in heart rate. Similar results were seen in a crossover study of chronic heart failure with reduced ejection fraction (HFrEF), where MBF increased from 0.6 ± 0.21 to 0.70 ± 0.20 in HFrEF patients and from 0.81 ± 0.27 to 1.05 ± 0.34 in their age-matched controls [9]. In both groups, the increase in MBF was independent of changes in myocardial energy expenditure.

Another study looked at the effects of hyperketonemia in 12 adults with heart failure (EF < 50%) and type 2 diabetes mellitus (T2DM) [49]. They observed that MBF (1.23 ± 0.09 vs. 1.18 ± 0.11 mL/min, *p* = 0.76) and myocardial glucose uptake (9.60 ± 1.01 vs. 8.56 ± 0.74 µmol/(min·100 g) were not changed after 6 h of bOHB infusion, with plasma levels of bOHB reaching 1.6 ± 0.2 mmol/L. Instead, MBF significantly increased in six subjects (“responders” 1.05 ± 0.13 to 1.38 ± 0.17, *p* = 0.003), and MBF significantly decreased in six subjects (“non-responders” 1.44 ± 0.07 to 0.95 ± 0.08, *p* = 0.007), which was found to correlate to high and low EF, respectively. Additionally, the study found that bOHB infusion increases cardiac output, stroke volume, and EF. This study suggests that baseline EF significantly correlates with response to MBF, such that subjects with low EF are less responsive to MBF. As discussed later, patients with isolated HFrEF responded to bOHB with improved cardiac energetics. Thus, the impaired metabolic phenotype seen in T2DM and severe HF may prevent the beneficial effects of KBs.

Other studies have also demonstrated that KBs increase cerebral and renal blood flow by up to 30% and 20%, respectively [50,51]. Thus, it can be concluded that in clinical models, bOHB administration leads to a robust increase in myocardial blood flow and alters metabolic substrate use, which is in line with the theory of metabolic reprogramming leading to improved perfusion in the heart. Recent work in the mouse has replicated the hyperemic response following acute bOHB administration. Thus, work in preclinical models may serve to further investigate the effect, safety, and underlying mechanism of bOHB administration on perfusion.

**Table 1:** Summary of clinical findings of KB effect on myocardial perfusion, peripheral vascular perfusion, and endothelial cell proliferation. bOHB (beta-hydroxybutyrate), CO (cardiac output), EDV (end diastolic volume), LVEF (left ventricular ejection fraction), IDDM (insulin-dependent diabetes mellitus), MAP (mean arterial pressure), MBF (myocardial blood flow), MEE (myocardial external energy efficiency), MVO_2_ (myocardial volume oxygen consumption), RPF (renal plasma flow), SV (stroke volume), SVR (systemic vascular resistance).

Author	Subjects (N)	Ketone Body	Outcome
Gormsen et al. [8] 2017	8 human subjects	Na-beta-hydroxybutyrate infusion (390 min) infusion vs. saline	Changes observed in hyperketonemia arm: -Myocardial glucose uptake reduction by half (304 ± 97 nmol/g/min [saline] vs. 156 ± 62 nmol/g/min [ketone], *p* < 0.01) -Increased heart rate by ~25% -Increase myocardial blood flow by ~75%
Nielsen et al. [9] 2019	10 HFrEF and 10 healthy subjects	3-h bOHB or placebo infusion: sodium-bOHB at a 7.5% concentration; glucose (20% solution, 60 mM KCl); low-dose insulinemic euglycemic clamp (0.3 IE insulin kg^−1^ h^−1^); randomized, single-blinded crossover study	Changes observed from before to after bOHB infusion: -MBF increased in both study groups during bOHB infusion, but slightly more in age-matched volunteers -SV increased in proportion to MVO2 -CO and HR increased -MEE was unchanged -MAP and SVR decreased
Solis-Herrera et al. [49] 2023	12 subjects with T2DM and HF (EF < 50%)	(I) 6-h bOHB infusion: Prime = 0.4 mg/kg/min for 20 min followed by constant rate = 0.2 mg/kg/min (II) 6 h bOHB infusion w/HCO3 control: Prime = 1.5 mg/kg/min for 20 min followed by constant rate = 0.75 mg/kg/min (III) 3-h bOHB infusion: Prime = 4.0 mg/kg/min for 20 min followed by constant rate = 2.0 mg/kg/min	(I) Plasma bOHB: 0.7 ± 0.3 (II) Plasma bOHB: 1.6 ± 0.2 -no change in MBF (1.23 ± 0.09 vs. 1.18 ± 0.11 mL/min, *p* = 0.76) MBF significantly increased in six subjects (“responders” 1.05 ± 0.13 to 1.38 ± 0.17, *p* = 0.003) -no change in MBF (1.23 ± 0.09 vs. 1.18 ± 0.11 mL/min, *p* = 0.76) MBF significantly decreased in six subjects (“non-responders” 1.44 ± 0.07 to 0.95 ± 0.08, *p* = 0.007) -CO, EF, SV increased (III) Plasma bOHB: 3.2 ± 0.2 mmol/L -CO, EF, SV increased
Svart et al. [51] 2018	9 human subjects	D,L-3-hydroxybutyric acid (75 g bOHB/L over four hours at a rate of 0.22 g/kg/h) vs. isotonic saline	At bOHB concentration of 5.5 ± 0.4 mmol/L: -Cerebral glucose utilization decreased by 14% -Cerebral blood flow increased 30%
Fioretto et al. [50] 1987	11 healthy human subjects and 11 patients with IDDM	D,L-3-hydroxybutyric acid infusion (40 μmol/kg/min and 30 μmol/kg/min for 180 min)	40 μmol/kg/min group: -Healthy—increased RPF (from 588 ± 78 to 706 ± 129 mL/min/1.73 m^2^) -IDDM—increased RPF (from 671 ± 101 to 781 ± 99 mL/min/1.73 m^2^) 30 μmol/kg/min increased RPF to a lesser extent than the 40 μmol/kg/min dose
Homilius et al. [36] 2023	Male Sprague Dawley rats	Na-beta-hydroxybutyrate infusion (390 min)	In vivo bOHB [2–4 mM]: -increased CO (by 28.3 ± 7.8%), SV (by 22.4 ± 6.0%), and LVEF (by 13.3 ± 4.6%) -decreased SVR (by 30.6 ± 11.2%) Ex vivo bOHB [10 mM]: -increase coronary perfusion (by 20.2 ± 9.5%]
Gopalasingam et al. [52] 2024	Danish Landrace × Yorkshire pigs	3-h infusion of D, L, or racemic mixture of b-OHB vs. isovolumic control	-D/L-bOHB and L-bOHB increased CO by 2.7 L/min -D-bOHB increased CO nonsignificantly -D/L-bOHB and L-bOHB reduced arterial elastance (afterload) -end-systolic elastance (contractility) decreased in L-bOHB -EDV (preload) decreased in D/L-bOHB -isolated coronary arteries, D- and L-bOHB dilated coronary arteries equally at concentrations 3 mmol/L

### 4.2. Preclinical Findings

The effect of KBs in non-human subjects has been well documented to promote peripheral artery vasodilation and myocardial blood flow. In the coronary vessel, the cardiac changes in response to bOHB vary amongst species and bOHB enantiomers [36,52,53]. D-bOHB has been found to have greater retention in heart tissue compared with L-enantiomer, which would be in line with D-bOHB having more of an oxidative role in heart tissue. Meanwhile, L-bOHB resulted in a greater increase in cardiac output due to a decrease in afterload in swine. These differences were hypothesized to be from higher circulating bOHB with L-bOHB infusion due to its slower metabolism at the whole-body level. When looking at the impact of enantiomer configuration on vasodilation, no differences were observed, as both enantiomers induced vasorelaxation at 3 mmol/L in preconstricted coronary arteries of swine [52]. Meanwhile, rat studies determined that bOHB increased coronary perfusion by up to 20% through direct vasorelaxation at supratherapeutic levels (>10 mmol) but had a modest vasodilatory effect at physiological levels (<3 mmol). bOHB infusion additionally increased cardiac output and stroke volume, and these results were consistent with and without endothelium [36]. Recently, a study in mice found that bOHB administration increased perfusion, though not from direct VSM vasorelaxation, as bOHB up to 5 mM did not induce changes in the diameter of isolated coronary arteries (<200 μm). Instead, they demonstrated that the hyperemic effects of bOHB required intact metabolic pathways in the cardiomyocytes, as knocking out BDH1 in cardiomyocytes abolished the hyperemic response to bOHB [38]. As discussed before, regulation of MBF is essential to maintaining heart function during rest, stress (exercise), and disease (ischemia–reperfusion, heart failure, aging). Therefore, the administration of bOHB is being investigated for its role in exercise capacity and potential as a therapeutic agent in heart disease.

## 5. Impact of Altered Myocardial Blood Flow on Ketone Body Utilization

### 5.1. Ischemia–Reperfusion

During ischemia, reduced coronary blood flow leads to decreased oxidative phosphorylation, resulting in shifts in myocardial energy substrate utilization. Upon reperfusion, fatty acid β-oxidation accelerates more rapidly than carbohydrate oxidation, leading to the uncoupling of glycolysis from glucose oxidation and further proton accumulation. This exacerbates metabolic inefficiency and impairs myocardial recovery. Studies have shown that stimulating glucose oxidation while inhibiting fatty acid oxidation improves myocardial efficiency during reperfusion and facilitates better functional recovery, underscoring the therapeutic potential of modulating substrate utilization to improve post-ischemic cardiac outcomes [54,55].

**Figure 3:**
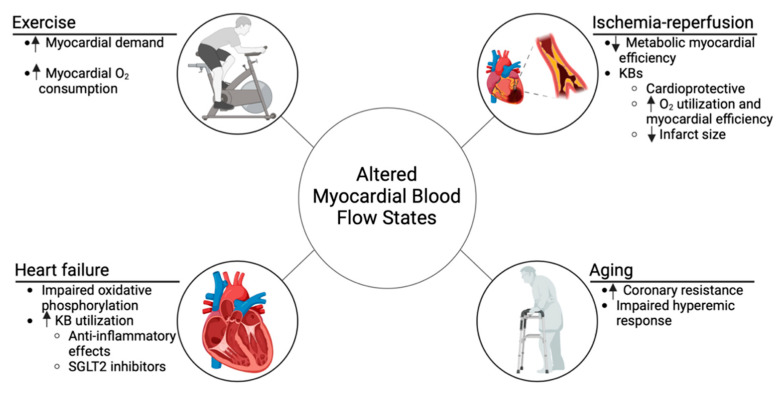
Physiological and metabolic changes under altered myocardial flow conditions: Certain states such as exercise, aging, heart failure, and ischemia–reperfusion are characterized by pathologic or physiological changes in myocardial flow. Exercise and aging may potentially benefit from KBs, while KBs have demonstrated benefits in heart failure and ischemia–reperfusion. KBs—ketone bodies. (upward arrow—increasing) (downward arrow—decreasing).

In this context, KBs offer a novel approach to modulating substrate metabolism. By promoting the coupling between glycolysis and glucose oxidation, KBs improve oxygen utilization during reperfusion, enhancing myocardial efficiency (Figure 3). This metabolic shift also spares oxygen—a crucial factor during ischemia and early reperfusion—supporting myocardial recovery.

Animal studies have demonstrated that KBs accumulate in the myocardium during low-flow ischemia, and human studies following acute myocardial infarction have reported elevated KB levels postreperfusion. For instance, in a 2021 study involving 369 patients with ST-segment elevation myocardial infarction, elevated circulating KBs at 24 h postreperfusion (median, 206 μmol per liter) were associated with larger myocardial infarct sizes (β = 1.56 per 100 μmol per liter; *p* = 0.016) and lower left ventricular ejection fraction (β = −1.78; *p* = 0.012) at a 4-month follow-up, suggesting a complex relationship between ketone metabolism and myocardial injury [56]. Post-ischemic elevations in endogenous KB levels are likely driven by the stress response to myocardial infarction or increased sympathetic activation.

Preclinical studies have shown that exogenous administration of bOHB before and during reperfusion reduces infarct size and improves cardiac function. These cardioprotective effects are thought to result from enhanced mitochondrial ATP production, reduced oxidative stress, and inhibition of the NOD-like receptor protein 3 (NLRP3) inflammasome, which limits the activation of proinflammatory cytokines such as interleukin-1β and interleukin-18. Additionally, bOHB’s antioxidant properties reduce reactive oxygen species production, potentially stabilizing atherosclerotic plaques and mitigating ischemia–reperfusion injury [57].

In human studies, the administration of exogenous KBs has shown similar cardioprotective effects. In one study involving 20 healthy participants, oral administration of KBs led to significant improvements in left ventricular ejection fraction (an increase of 3.1%; *p* < 0.001), global longitudinal strain (an increase of 2.0%; *p* < 0.001) and left atrial reservoir strain (an increase of 7%; *p* = 0.005). Although myocardial perfusion was not directly measured, the observed reductions in systemic vascular resistance and improvements in ventricular function suggest enhanced perfusion [58]. Although promising cardioprotective effects of KBs have been demonstrated in preclinical models, further human studies are needed to assess the direct impact of exogenous KB administration on coronary blood flow and myocardial perfusion, particularly in ischemic heart disease.

### 5.2. Heart Failure

HF, whether characterized by HFrEF or preserved ejection fraction (HFpEF), involves significant alterations in myocardial metabolism and perfusion. In HFrEF, defined by an ejection fraction below 40%, impaired cardiac output and increased ventricular pressures lead to reduced MBF and coronary perfusion deficits, compounded by microvascular dysfunction and endothelial injury. Conversely, HFpEF, defined by an ejection fraction of 50% or greater, typically maintains normal MBF at rest; however, during exertion, increased myocardial stiffness and impaired vasodilation can lead to subendocardial ischemia [59].

In both forms of HF, there is a metabolic shift toward increased KB utilization (Figure 3). Due to mitochondrial dysfunction and impaired oxidative phosphorylation, the myocardium becomes more reliant on ketones such as bOHB and acetoacetate, which provide a more efficient energy substrate. bOHB offers a higher ATP yield per unit of oxygen compared to fatty acids, which is critical for energy-starved myocardial tissue. Additionally, bOHB has anti-inflammatory properties, primarily through inhibition of the NLRP3 inflammasome, further conserving myocardial energy and mitigating damage [60].

Supporting the complexity of KB metabolism in HF, a longitudinal study involving 1382 patients with HF identified a significant association between elevated plasma KB levels and advanced HF, particularly in those with HFpEF [61]. This study found that higher KB levels were linked to increased mortality risk (hazard ratio, 1.23), prompting critical questions regarding whether elevated ketones contribute to HF progression or merely reflect disease severity [61]. Further insights into cardiac metabolism demonstrated increased uptake of free fatty acids and ketone bodies in patients with aortic stenosis-induced left ventricular hypertrophy, suggesting that these metabolic adaptations could present novel therapeutic targets [62].

SGLT2 inhibitors have become a pivotal advancement in HF management, reducing hospitalizations and mortality across the spectrum of HF, irrespective of ejection fraction. Their broad therapeutic impact is attributed to a combination of natriuretic, hemodynamic, and anti-inflammatory effects, as well as their metabolic influence, which shifts substrate metabolism toward fatty acid oxidation and increases bOHB levels, promoting KB production. This shift enhances cardiac energetics and contributes to the broader therapeutic impact of SGLT2 inhibitors [63]. Exogenous ketones, particularly bOHB salts, and esters have shown potential as therapeutic agents for HF by offering an alternative energy source to improve myocardial energetics. Studies in HFrEF (Table 2) indicate that these supplements enhance cardiac output and LV function by reducing reliance on impaired glucose and fatty acid oxidation pathways.

A study assessed maximal ketone body utilization in patients with HFrEF and demonstrated a 12-fold increase in serum bOHB and a higher fractional extraction of bOHB that was inversely correlated with left ventricular ejection fraction (LVEF) [64]. This study showed a correlation between bOHB extraction in HFrEF patients and the degree of cardiac dysfunction. Furthermore, two studies assessed patients with cardiogenic shock and decompensated HF requiring inotropic support, respectively. Subjects in cardiogenic shock who were administered KB showed improved cardiac output, LVEF, and right and left heart filling pressures [65]. Likewise, subjects with HFrEF receiving inotropic support had a 39% increase in cardiac index after bOHB administration [66]. Research has also shown significant improvements in LVEF, cardiac output, and stroke volume of HFrEF and T2DM patients treated with incremental doses of bOHB [67].

Conversely, one study found that bOHB infusion in heart failure with T2DM improved LV function without altering myocardial glucose uptake [49]. Another study assessing the effects of KBs on cardiac energetics in T2DM, HFrEF, and healthy subjects found no improvements in LVEF or MBF across all study groups [68]. These findings highlight the variability in responses to ketone therapy, prompting further investigation into its potential benefits.

Recent work in the mouse has replicated the hyperemic response following acute bOHB administration. Thus, work in preclinical models may serve to further investigate the effect, safety, and underlying mechanism of bOHB administration on perfusion. A recent randomized, double-blind, crossover trial focused on ketone ester treatment in 24 patients with HFpEF and T2DM. After two weeks of ketone ester treatment (25 g of D-beta-hydroxybutyrate–(R)-1,3-butanediol four times daily), right heart catheterization and echocardiography revealed increased cardiac output (increase of 0.2 L per minute; 95% CI, 0.1 to 0.3) and reduced pulmonary capillary wedge pressure both at rest (decrease of 1 mm Hg; 95% CI, −2 to 0) and during peak exercise (decrease of 5 mm Hg; 95% CI, −9 to −1). These results suggest that KB treatment may provide a new modality for managing HFpEF, particularly in diabetic patients [69]. These findings underscore the possible therapeutic role of KB modulation in HF, particularly by using SGLT2 inhibitors and ketone ester treatments. However, further research is imperative to fully delineate the long-term effects and optimize therapeutic strategies involving KBs in HF management.

**Table 2:** Human clinical studies on the role of exogenous ketones in ATP (adenosine triphosphate), AUC (area under the curve), HFrEF (heart failure with reduced ejection fraction), bOHB (beta-hydroxybutyrate), GLS (global longitudinal strain), KBs (ketone bodies), KE (ketone ester), LVEF (left ventricular ejection fraction), PET (positron emission tomography), T2DM (type 2 diabetes mellitus).

Study	Aim	Methodology	Outcome
Monzo et al. [64] (2021)	Assessed the maximal KB utilization capacity in HFrEF patients compared to controls, with a focus on the effect of oral KE administration.	19 HFrEF patients and 9 controls underwent arterial and coronary sinus sampling to measure substrate and oxygen extraction. In a separate experiment, 11 HFrEF patients and 6 controls were given 25 g of oral KE, with measurements taken 80 min post-administration to assess ketone utilization.	bOHB levels increased 12.9-fold after KE administration. HFrEF patients showed higher fractional extraction (52%) compared to controls (39%, *p* = 0.035). bOHB fractional extraction correlated with bOHB delivery (r = 0.90), LV mass (r = 0.56), LV diameter (r = 0.65), and inversely with LVEF (r = −0.59), all *p* < 0.05.
Hansen et al. [70] (2024)	Assessed the effects of 14-day KE treatment on resting and exercise hemodynamics in HFrEF patients.	24 patients with HFrEF participated in a randomized, double-blind, crossover design. Each patient received 14 days of KE treatment or an isocaloric comparator, with a 14-day washout period. Hemodynamic and echocardiographic assessments were performed after each treatment phase.	KE treatment increased resting cardiac output by 0.3 L/min (5.2 vs. 5.0 L/min) and reduced pulmonary capillary wedge pressure by 2 mmHg (8 vs. 11 mmHg). LVEF improved by 3% (37% vs. 34%), and NT-proBNP levels decreased by 18% (98 ng/L).
Sramko et al. [66] (2022)	Investigated the feasibility, safety, and acute hemodynamic effects of BHB administration in HFrEF patients receiving inotropic support.	8 patients with decompensated heart failure on inotropic support received 75 g of oral bOHB over 24 h in 3 h intervals. Hemodynamics were measured using Swan–Ganz catheterization and serum bOHB concentrations were assessed before and after administration.	bOHB increased cardiac index by 39% within 3 h (from 2.5 ± 0.5 to 3.4 ± 0.8 L/min/m^2^, *p* = 0.003), with a peak increase of 52% (1.2 ± 0.4 L/min/m^2^) observed after 5 h. Mild ketosis was induced (1.8 ± 0.6 mmol/L), with no significant complications reported.
Kotha et al. [68] (2024)	Evaluated the effects of short-term exogenous KE on cardiac energetics, function, and myocardial lipid content in patients with T2DM, HFrEF, and healthy volunteers.	87 participants (33 T2DM, 29 HFrEF, and 25 healthy volunteers) received 2 weeks of daily KE supplementation (30 g/day). Cardiac function and energetics were assessed via blood tests, MRI, and spectroscopy.	T2D patients showed an increase in the phosphocreatine/ATP ratio from 1.6 to 1.9 (*p* = 0.01), with no significant changes in HFrEF or healthy participants. No improvements were noted in LVEF, GLS, myocardial blood flow, or lipid content across all groups.
Hansen et al. [65] (2023)	Evaluated the hemodynamic effects of a single dose of exogenous KE in patients with cardiogenic shock.	12 patients with cardiogenic shock were randomized in a double-blind, crossover design to receive an enteral bolus of KE or isocaloric placebo. Hemodynamics were assessed over 3 h with pulmonary artery catheterization, echocardiography, and blood tests.	KE increased circulating bOHB to 2.9 ± 0.3 mmol/L (*p* < 0.001), improved cardiac output by AUC of relative change of 61 ± 22 L (*p* = 0.044), and increased LVEF by 4% (*p* = 0.005). KE also reduced right (*p* = 0.048) and left (*p* = 0.017) ventricular filling pressures and enhanced forearm perfusion by 3% (*p* = 0.026).
Solis-Herrera et al. [67] (2022)	Investigated the mechanism behind SGLT2 inhibitors by examining the effects of elevated ketone levels on left ventricular function and myocardial glucose uptake in patients with T2DM and HFrEF.	36 patients with T2DM and HFrEF (LVEF < 45%) were divided into three groups. Each group received increasing doses of bOHB or NaHCO_3_ (control), with cardiac MRI and PET used to assess cardiac function and metabolism.	Plasma ketone levels increased to 1.0, 1.3, and 2.5 mmol/L in Groups I, II, and III, respectively. Higher ketone levels improved cardiac output, ejection fraction, and stroke volume, with Group III showing the greatest improvement in LVEF (*p* < 0.001). Myocardial glucose uptake was unchanged, suggesting ketones provided an additional fuel source without altering glucose metabolism.

### 5.3. Aging

As life expectancy continues to increase, the effects of aging on vascular health become more pronounced, amplifying the risk of cardiovascular disease [71]. Several studies have indicated that the incidence of arterial stiffness and hypertension is especially high in older populations [72]. The development of vascular rigidity is influenced by various processes such as metabolic dysfunction, inflammation, and neurohumoral dysregulation, which can impair endothelial wall integrity through mechanisms involving matrix metalloprotease activity, increased collagen accumulation, calcification, and reduced elastin levels [72,73]. The cumulative impact of increased systemic vascular stiffness would no doubt influence myocardial perfusion, a process mainly dictated by vasodilation of coronary vessels.

Early studies evaluating hemodynamic differences in elder (63 ± 8 years) and young (31 ± 9 years) populations reported that baseline rate–product pressure (RPP) (6895 ± 1070 vs. 8634 ± 1890; *p* < 0.01) and MBF (0.92 ± 0.25 vs. 0.76 ± 0.17 mL·min^−1^·g^−1^; *p* < 0.05) were higher in the aged group [74]. Similarly, although not significantly different, dipyridamole-induced hyperemia trended lower among aged populations (3.06 ± 0.76 vs. 2.64 ± 0.58 mL·min^−1^·g^−1^), which, in combination with the observed higher baseline MBF, may explain the reported myocardial flow reserve being lower in the aged group (4.1 ± 0.9 vs. 3.0 *±* 0.70; *p* < 0.0001) [74]. Notably, coronary resistance was also higher in the aged group (38 ± 10 vs. 31 ± 8 mmHg·mL^−1^·g^−1^·min^−1^; *p* < 0.05) [74].

Subsequent studies have seen mixed results on baseline MBF, while all have seen impaired hyperemic response with increasing age. One particular study observed, through dynamic PET, no differences in baseline MBF but recapitulated the finding that older subjects had a reduced hyperemic response and lowered myocardial perfusion reserve, suggesting a blunted blood flow response to increasing work demands [17,75]. More recent efforts using CMR evaluated MBF in 151 healthy volunteers (19 to 79 years of age) and found that resting MBF negatively correlated with increasing age (*r* = −0.337, *p* < 0.001) when corrected for RPP, which also appeared to correlate with aging (*r* = 0.247, *p =* 0.004) [76]. In another study, adenosine-induced hyperemia was impaired, as shown by reduced MBF and myocardial perfusion reserve being negatively correlated with increasing age (r = −0.43, *p* < 0.001, and r = −0.34, *p* < 0.001, respectively), collectively suggesting that higher cardiac work may be necessary to match increasing stress demands in advanced age [76]. Lastly, with increasing age, it has been thought that myocardial substrate utilization may be significantly altered [77,78]. Specifically, it has been reported that fatty acid oxidation and glucose utilization are impaired in aged hearts [77,79,80]. As such, alternative fuel sources like ketones may serve as essential substrates for cardiac energy provision. However, there have been no studies directly evaluating cardiac ketone oxidation in aging, although it has been shown that plasma b-hydroxybutyrate is not different between aged and young mice [80]. As discussed earlier, changes in cardiac substrate utilization are sufficient to alter baseline coronary flow independent of altered cardiac work, although the underlying mechanism remains unknown. As such, the altered substrate uptake in aged hearts could be a key factor in the reductions of both resting and hyperemic myocardial flow.

### 5.4. Exercise

During exercise, there is a rapid increase in myocardial metabolic demand, which initiates compensatory mechanisms to boost cardiac oxygen availability. At rest, the heart exhibits an exceptionally high oxygen extraction rate (~70–80%) due to its dense capillary network, limiting further increase during exercise [12,27]. Therefore, the primary means for meeting the increased oxygen demand is through enhanced MBF by decreasing coronary vascular resistance [27,81]. Seminal studies in dogs by von Restorff et al. reported that heavy treadmill exercise elicited a 434% increase in coronary blood flow, which coincided with an increase in myocardial oxygen consumption (rest = 0.09 ± 0.01, exercise = 0.57 ± 0.05 mL·min^−1^·g^−1^) [82]. Notably, several mechanisms have been proposed to regulate MBF during exercise including autonomic regulation and local metabolites [27].

With increasing myocardial oxygen extraction and utilization during exercise, changes in local metabolites have been theorized to be major regulators of coronary flow. However, isolated evaluations of nitric oxide, prostanoids, endothelin, adenosine, and K_ATP_ channel opening in the context of exercise-induced hyperemia have been inconclusive, with many studies reporting that individual modulation of these factors may not regulate MBF during exercise [27]. A simultaneous blockade of multiple metabolic factors, including adenosine receptors, NO synthase, and K_ATP_ channels, has also shown conflicting results, collectively suggesting that in vivo regulation of coronary flow during exercise may be supported by several layers of redundancy [83,84].

Circulating ketone concentrations have been reported to have increased following prolonged exercise, rising from 100–250 µM to approximately 1 mM in adult humans [85]. As such, there has been immense interest in exploring the potential role of ketone utilization on exercise performance. However, the current literature on exogenous ketone supplementation as it relates to exercise performance has been largely inconsistent [86]. This may be partially explained by the variation in the type of KB supplement used or exercise performed. One of the few studies demonstrating a notable impact of exercise capacity reported that ketone–ester supplementation, coupled with carbohydrates, may improve bicycle ergometer time performance by ~2% [10]. To date, it is not known whether exogenous ketone supplementation influences cardiac efficiency and MBF during exercise, although acute infusion of bOHB has been seen to improve MBF and CO, both of which are important for supporting increased cardiac workload [8].

Preclinical studies on rodents suggest that KB + glucose supplementation increases cardiac efficiency by 28% (J/mol O_2_ consumed). It is important to consider here that these isolated heart studies may not recapitulate the in vivo system, especially under exercise conditions.

### 5.5. Unresolved Questions

As research on the regulation of MBF expands, evidence continues to grow supporting local metabolic changes as a primary regulator of MBF in the autoregulatory pressure range. Yet the long-standing question of how MBF and local metabolic changes are so tightly coupled remains a central question of coronary physiology. Multiple byproducts of substrate metabolism such as lactate, hydrogen peroxide, adenosine, and nitric oxide have been noted as vasoactive. Additionally, changes in redox states, electrical conductance, and autonomic signaling may also influence VSM constriction/relaxation [22]. What is known is that altering substrate availability and metabolic reprogramming can profoundly impact vasoactivity in the heart. Therefore, the next steps are to measure specific changes to the local environment of the heart based on substrate availability and whether the substrates need to be metabolized to produce metabolic vasodilation/constriction.

Additionally, more research needs to be conducted on when metabolic supplementation is beneficial, as some groups appear to be metabolically sensitive and able to respond to interventions like KB supplementation, while others appear immune. This could be due to underlying metabolic expression of disease states or underlying genetic differences. Regardless, exogenous ketones such as bOHB may require certain pathways in the cell to remain intact to improve cardiac function. Understanding what pathways in the cell are essential to increasing perfusion will be an essential next step in understanding the local metabolic hypothesis and leveraging it to enhance perfusion.

## 6. Strengths and Limitations

The studies discussed in this review were collected in a non-standardized and non-systematic manner. Consequently, there is a potential for bias. Though the studies included in this review were published over a ~40-year range, human subject research is lacking in some areas. While several studies have been conducted on human subjects regarding the effect of KBs on myocardial function in HFrEF, little is known about their effects in healthy participants, during exercise, and in elderly populations. The available human studies evaluated also had small sample sizes, which may lead to bias and misinterpretation of results. Nevertheless, the summative approach of this review supported a comprehensive discussion on the potential roles and potential mechanisms of increased MBF during acute hyperketonemia. To our knowledge, no other review articles have been published over the past 20 years summating the impact and mechanisms of KBs on myocardial perfusion. Our review identified areas for future investigations, including KBs influence on MBF during exercise in healthy human subjects and in the elderly.

## 7. Conclusions

In conclusion, optimal MBF during rest and periods of increased demand is essential for cardiac health, while a mismatch between blood supply and demand is associated with cardiovascular disease. There is growing support that hyperketonemia not only improves cardiac function but also induces a significant increase in MBF. It is important to note that this response was limited in individuals with underlying metabolic diseases, such as diabetes. Furthermore, bOHB administration in isolated vessels did not induce direct vasodilation. Thus, ketones indirectly generate a vasoactive response. This aligns with the theory that local changes in coronary metabolism serve as a major regulator of coronary flow. Therefore, advancing research into the potential cardiac benefits of KB administration and their underlying mechanisms could lead to novel therapeutic avenues in cardiac health (athletes) and disease (aging, heart failure). This will also shed light on one of the long-standing questions in coronary physiology. Most studies discussed in this review support the therapeutic benefits of bOHB in HFrEF and HFpEF patients, with a few showing no benefit in healthy and HF subjects. Some studies suggest that bOHB improves cardiac output/index, ejection fraction, and cardiac filling pressures in HF patients. Likewise, the main clinical study assessing KBs in healthy participants showed increased MBF with KB administration. Overall, the therapeutic potential of bOHB is promising for heart failure and warrants further investigation into its impact on exercise capacity, myocardial ischemia, and aging.

## Abbreviations

The following abbreviations are used in this manuscript:

bOHB	beta-hydroxybutyrate
FFA	Free fatty acid
HFpEF	Heart failure with preserved ejection fraction
HFrEF	Heart failure with reduced ejection fraction
KB	Ketone bodies
LVEF	Left ventricular ejection fraction
MCT	Monocarboxylate transporters
MBF	Myocardial blood flow
MRI	Magnetic resonance imaging
MVO_2_	Myocardial volume oxygen consumption
NLRP3	NOD-like receptor protein 3
PET	Positron emission tomography
RPP	Rate–product pressure
SGLT2	Sodium–glucose cotransporter 2
T2DM	Type 2 diabetes mellitus
VSM	Vascular smooth muscle
